# Donor–Acceptor–Donor 1*H*-Benzo[*d*]imidazole Derivatives as Optical Waveguides

**DOI:** 10.3390/molecules28124631

**Published:** 2023-06-08

**Authors:** Carlos Tardío, Javier Álvarez Conde, Ana María Rodríguez, Pilar Prieto, Antonio de la Hoz, Juan Cabanillas-González, Iván Torres-Moya

**Affiliations:** 1 Department of Inorganic, Organic Chemistry and Biochemistry, Faculty of Chemical Science and Technologies, University of Castilla-La Mancha-IRICA, 13071 Ciudad Real, Spain; carlos.tardio@uclm.es (C.T.); anamaria.rfdez@uclm.es (A.M.R.); mariapilar.prieto@uclm.es (P.P.); 2 Madrid Institute for Advanced Studies, IMDEA Nanociencia, Calle Faraday 9, Ciudad Universitaria de Cantoblanco, 28049 Madrid, Spain; javier.alvarez@imdea.org; 3 Department of Organic Chemistry, Faculty of Chemical Sciences, Campus of Espinardo, University of Murcia, 30010 Murcia, Spain

**Keywords:** optical waveguide, 1*H*-benzo[*d*]imidazole, donor-acceptor-donor systems

## Abstract

A new series of donor–acceptor–donor (D–A–D) structures derived from arylethynyl 1*H*-benzo[*d*]imidazole was synthesized and processed into single crystals with the goal of testing such crystals’ ability to act as optical waveguides. Some crystals displayed luminescence in the 550–600 nm range and optical waveguiding behavior with optical loss coefficients around 10^−2^ dB/μm, which indicated a notable light transport. The crystalline structure, confirmed by X-ray diffraction, contains internal channels that are important for light propagation, as we previously reported. The combination of a 1D assembly, a single crystal structure, and notable light emission properties with low losses from self-absorption made 1*H*-benzo[*d*]imidazole derivatives appealing compounds for optical waveguide applications.

## 1. Introduction

In recent years, photonic integrated circuits (PICs) have become of paramount importance in many applications, such as optical signal processing, biomedical applications, and optical computing. Although the most common platforms for PICs are now based on inorganic bulk semiconductors such as InP or Si, alternatives such as 2D semiconductors [1], colloidal quantum dots [2], hybrid perovskites [3] and organic crystals [4] are the subjects of extensive research. 

Optical waveguide is a pivotal and fundamental component of PICs. This physical structure can conduct and propagate light efficiently with minimal loss. The two factors that influence the behavior of the optical waveguide are the total internal reflection and the fact that the medium must have an index of refraction higher than the external medium. Thus, when the incident light angle is higher than the critical angle, the light is confined and propagated along the structure, following the Snell law [5,6].

Organic crystals possess unquestionable advantages as light emitting and transporting media, including their capability to guide light in the visible region giving rise to large operational bandwidths, low transmission losses, simple and inexpensive processing into micro-/nanofibers of interest for miniaturization, high refractive indices, well-defined crystal faces with few grain boundaries and surface defects, and better heat dissipation [7,8,9]. Owing to their large chemical versatility, the waveguide geometry and transmission properties can be tailored through chemical functionalization of the molecular building blocks. For these reasons, in recent years there has been a large increase in the use of organic crystals as optical waveguides [10,11,12,13,14,15,16].

Integration of organic crystals in PICs remains a challenge. Nevertheless, extensive research is being carried out in this field to achieve such integration. For example, a PIC was achieved by using two different organic crystals in which, upon receiving the signal input, the circuit executed direction-specific optical outputs [17]. Furthermore, it is now possible to identify various real applications in which optical waveguides have already been successfully implemented, including highlighting the novel 5G technology with the employment of organic flexible crystals [18], use as media in optical communications [19], application in the construction of optical waveguide multimodal sensors for measuring human temperature [20], or as materials that possess the ability to convert light into mechanical work [21], demonstrating the relevance of this type of physical structure in the present and in the near future.

We recently described some examples of organic molecules with D–A–D architectures that show optical waveguiding behavior. They consist of an electron donor (D) and an electron acceptor (A) connected by a π-conjugated bridge [22], which facilitates intramolecular charge transfer (ICT) [23] and allows modulation of the energy bandgap, giving rise to luminescent optical waveguides that emit over a wide spectral range [22]. 

In this report, we extend this concept to the 1*H*-benzo[*d*]imidazole moiety, an interesting core, as it displays a strong acceptor character due to the presence of a C=N bond, which can be easily modulated with the introduction of different groups in positions 4 and 7. In addition, the NH group facilitates crystalline self-assembling via intermolecular hydrogen bonding, which is required for obtaining fibers or crystals with optical waveguiding behavior.

Despite these interesting properties, 1*H*-benzo[*d*]imidazole is still an unexplored moiety in material science, and only some examples of 1*H*-benzo[*d*]imidazole derivatives can be found, with applications as electrochromic materials [24], in organic electronics as photoactive components in organic solar cells [25] or OLEDs [26], and in gels [27].

**Figure 1:**
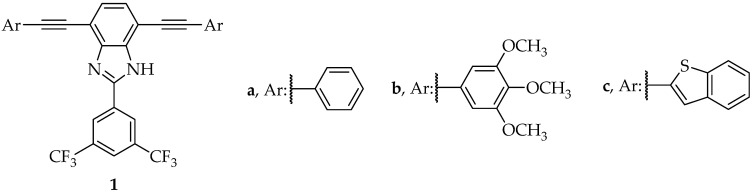
D–A–D 1*H*-benzo[*d*]imidazole derivatives (**1a**–**c**) synthesized.

Here, we describe three symmetrical D–A–D 2-(3,5-bis(trifluoromethyl)phenyl)-4,7-bis(arylethynyl)-1*H*-benzo[*d*]imidazoles (**1a**–**c**) (Figure 1) to study the optical waveguiding properties of 1*H*-benzo[*d*]imidazole-derived crystals and modulate the emission. 

Two electron-attracting trifluoromethyl groups were introduced in the 2-aryl group in order to increase the acceptor character of the benzimidazole moiety. In the same way, three different donor groups were introduced in positions 4 and 7 using alkynyl groups as linkers, as alkynyl groups facilitate the formation of supramolecular structures due to their ability to form CH-π interactions [22,28]. It should be noted that, to date, no 1*H*-benzo[*d*]imidazole-derived crystals with optical waveguide properties have been reported.

## 2. Results and Discussion

### 2.1. Preparation of 1H-Benzo[d]imidazole Derivatives ***1***

**Scheme 1:**
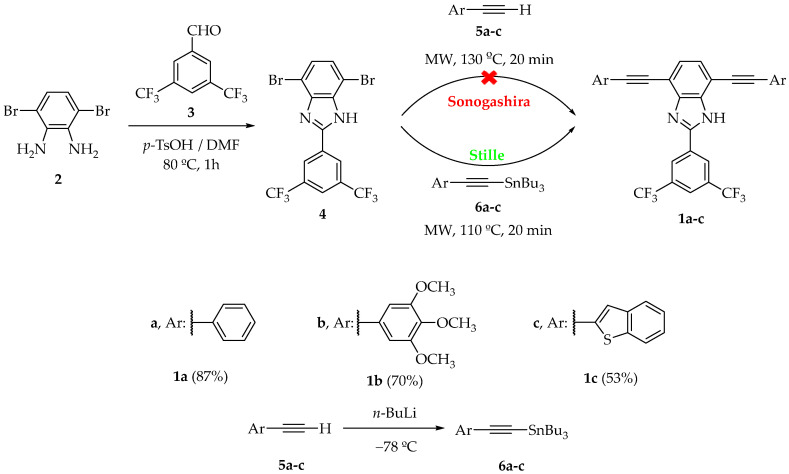
Synthetic procedure for the synthesis of D–A–D 1*H*-benzo[*d*]imidazole derivatives **1**.

1*H*-benzo[*d*]imidazole derivatives **1** were synthesized in two steps, as shown in Scheme 1. Thus, a mixture of 3,6-dibromobenzene-1,2-diamine (**2**) and 3,5-bis(trifluoromethyl)benzaldehyde (**3**) in the presence of *p*-toluenesulfonic acid resulted in the dibromo derivative **4** in 84% yield. Synthesis of the final bisarylethynyl-1*H*-benzo[*d*]imidazoles **1a**–**c** was attempted by a microwave-assisted Sonogashira C-C cross-coupling reaction between **4** and the corresponding alkynyl derivative **5**, as we previously described [22]. However, unreacted dibromo benzimidazole **4** was recovered in a complex mixture. Recently, it was stated that the presence of pyridinic or pyrrolic nitrogens and their different Cu(I) coordination may influence the activation of the triple bond [29]. Then, the basicity of the pyridinic nitrogen of the benzimidazole could also justify the lack of reactivity in the Sonogashira reaction. Derivative **1** was obtained in good yields (53–87%) using an alternative microwave-assisted Stille reaction between **4** and the corresponding arylethynyllstannane **6**. 

### 2.2. Theoretical Calculations

**Figure 2:**
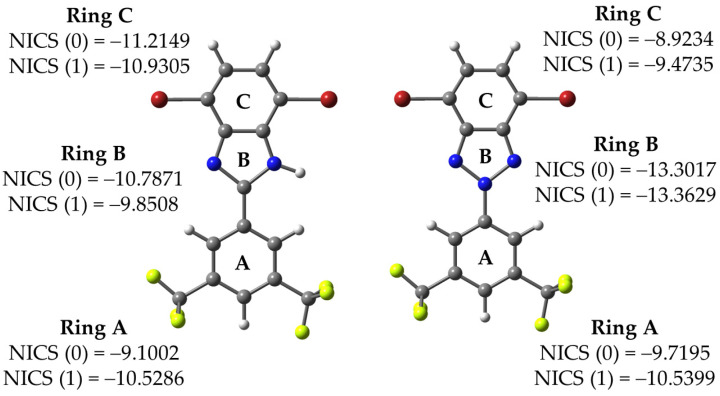
NICS(0) and NICS(1) values for compound **4** and dibrominated benzotriazole analogous computed at the B3LYP-D3/6-31G(d) level. Values are in ppm. Grey atoms are C, red atoms are Br, blue atoms are N, yellow atoms are F and white atoms are H.

A deeper understanding of the non-reactivity shown by **4** in Sonogashira reactions, as compared to analogous benzotriazole systems, has been achieved by analysis of the aromaticity of the rings within the DFT framework at B3LYP-D3/6-31G(d) level via the nucleus-independent chemical shift (NICS) method developed by Schleyer et al. [30]. NICS has been widely used as an aromaticity probe by computing the absolute magnetic shielding at the point of interest [31]. In order to have a complete overview, we calculated the NICS values at the ring-critical points and at 1 Å above as NICS(0) and NICS(1), respectively. The results obtained for the three aromatic rings of **4** are shown in Figure 2.

The NICS(0) and NICS(1) values obtained for ring C (−11.21 and −10.93 ppm, respectively) were more negative than the values described for analogous dibrominated benzotriazole (NICS(0) and NICS(1) values of –8.92 and –9.47 ppm, respectively) previously employed by our group [22]. Therefore, the NICS probe indicated a higher aromaticity of the dibrominated core in **4** compared to analogous dibrominated benzotriazole, in line with the lower reactivity of the former toward the Sonogashira coupling reaction (i.e., harder conditions are required for 1*H*-benzo[*d*]imidazole core, compared to benzotriazole analogous).

**Figure 3:**
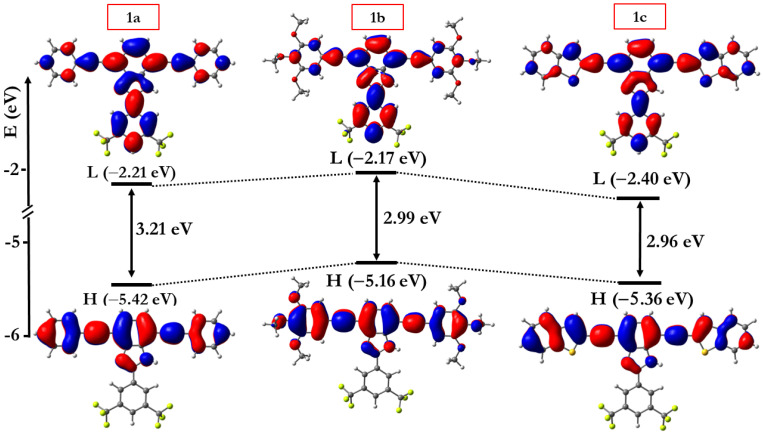
Topologies and energies of the HOMO (H) and LUMO (L) molecular orbitals of compounds **1a**–**c** obtained by DFT calculations at the B3LYP-6-31G(d,p) level. Grey atoms are C, red atoms are O, blue atoms are N, yellow atoms are F and white atoms are H.

On the other hand, with the aim of analyzing the molecular properties of the synthesized compounds, the minimum-energy optimized structures at B3LYP/6-31G(d,p) theoretical level were calculated. In all cases, the highest occupied molecular orbital (HOMO) was located mainly in the horizontal branch of the molecules, while the lowest unoccupied molecular orbital (LUMO) was located mainly in the vertical branch, with an overlap between both orbitals on the central acceptor core, which facilitates intramolecular charge transfer (ICT) from the electron-donating to the electron-withdrawing units. The calculated HOMO–LUMO energies, HOMO–LUMO gaps and topologies are shown in Figure 3.

Modification of the peripheral donor groups produced variations in the energy values of the frontier molecular orbitals, so that the HOMO–LUMO gap could be modulated by introducing different electron-donating groups. Thus, compound **1a**, which possesses weaker donor groups, showed a higher HOMO–LUMO gap value than derivatives **1b** and **1c**, with stronger donor groups and more effective π-conjugation.

### 2.3. Photophysical Data

**Figure 4:**
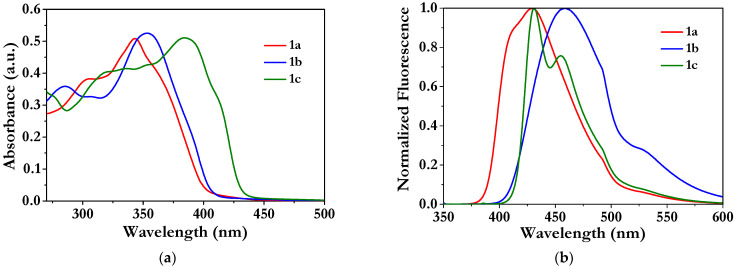
UV–Vis absorption (**a**) and photoluminescence (**b**) spectra of **1a**–**c** derivatives (298 K, CHCl_3_, 10^−5^ M). All PL spectra were measured upon photoexcitation at the maximum absorption peak of the corresponding compound.

The UV-vis and fluorescence spectra of derivatives **1,** recorded in 10^−5^ M CHCl_3_ solutions, are shown in Figure 4 and the photophysical values are summarized in Table 1. 

**1a**–**c** shows an absorption band ascribed to a π–π* transition (284–316 nm) and a dominant band at low energy (342–384 nm), ascribed to an intramolecular charge transfer (ICT), as supported by computational studies (Figure S1 and Table S1). Note that the theoretical results were in good agreement with the experimental results. Displacement of the absorption bands (λ_max_) followed the **1a** < **1b** < **1c** trend, which was related to the increase in the donor character of the peripheral groups. Thus, phenyl derivative (**1a**) showed an absorption band shifted to the blue, as a consequence of the weaker electron-donor character of the phenyl group, while **1b** (trimethoxyphenyl) and **1c** (benzothiophenyl), with higher donor character, resulted in a bathochromic shift of the maxima.

The fluorescence bands of **1a**–**c** were between 429–459 nm and followed the same trend as absorption. In contrast to the absorption maxima, **1b** showed a slightly larger shift of the emission band in comparison with **1c**.

In general, all compounds displayed moderate- to high-fluorescence quantum yields ranging from 0.55 to 0.78.

### 2.4. Molecular Self-Assembly

The generation of supramolecular aggregates was carried out by the slow diffusion technique. Thus, a vial containing a dilute solution (1 mg) of **1a**–**c** in 1 mL of tetrahydrofuran or chloroform, as good solvents, was gently introduced into a second vessel containing a poor solvent, such us hexane, acetonitrile, methanol, or ethanol. This second vessel was closed and, after seven days, the formation of supramolecular structures was observed.

**Figure 5:**
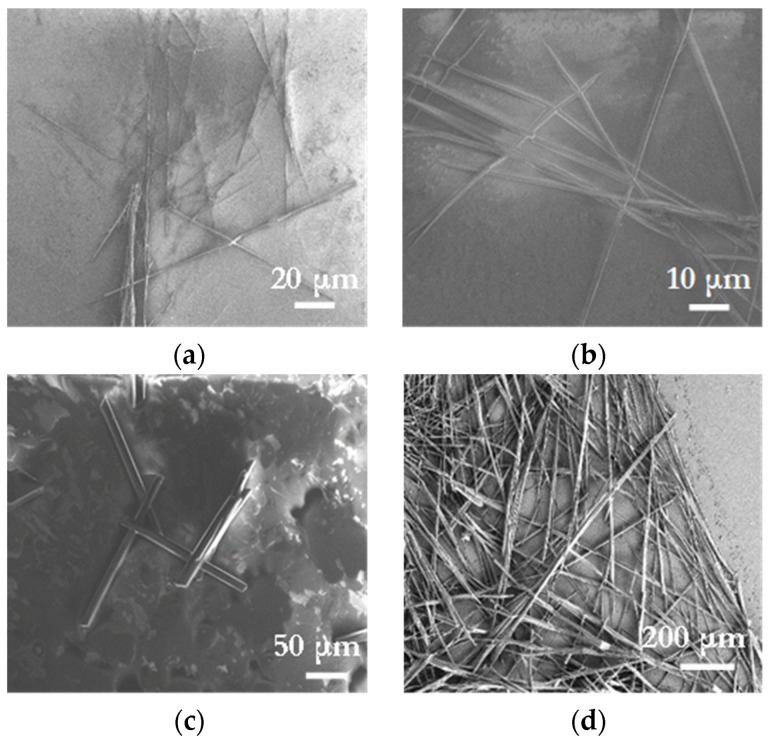
SEM images of the supramolecular aggregates of (**a**–**c**) **1b** (THF/MeOH, THF/EtOH, and CHCl_3_/CH_3_CN, respectively) and (**d**) **1c** (THF/EtOH).

**1a** did not lead to the formation of supramolecular structures in any of the mixtures used. In contrast, **1b** resulted in the formation of well-defined needle-shaped crystals using different solvent mixtures (Figure 5a–c), whereas **1c** only crystallized in THF/ethanol (Figure 5d). 

The thicknesses of the structures ranged from 5 to 20 μm and lengths ranged from tens to hundreds μm, in some cases reaching more than 1 mm in length.

### 2.5. Optical Waveguide Properties

**Figure 6:**
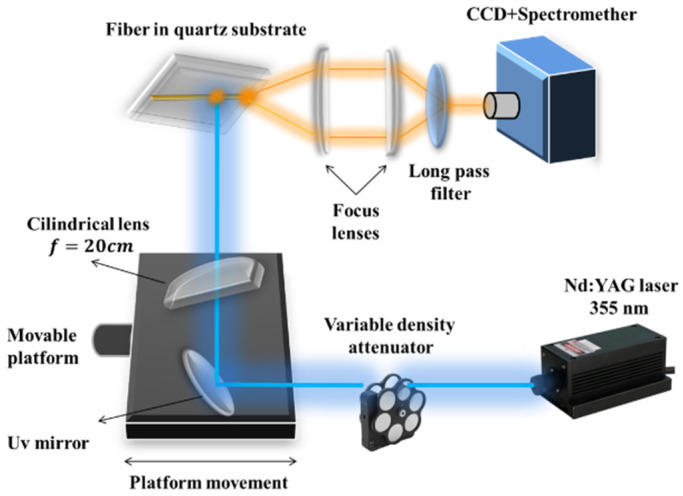
Schematic experimental setup employed for the optical waveguiding behavior measurements.

To elucidate their optical waveguide properties, crystals of **1b** and **1c** were studied by fluorescence microscopy in the experimental setup shown in Figure 6. Thus, the crystals were excited in the fiber body with a photoexcitation centered at 365 nm. 

**Figure 7:**
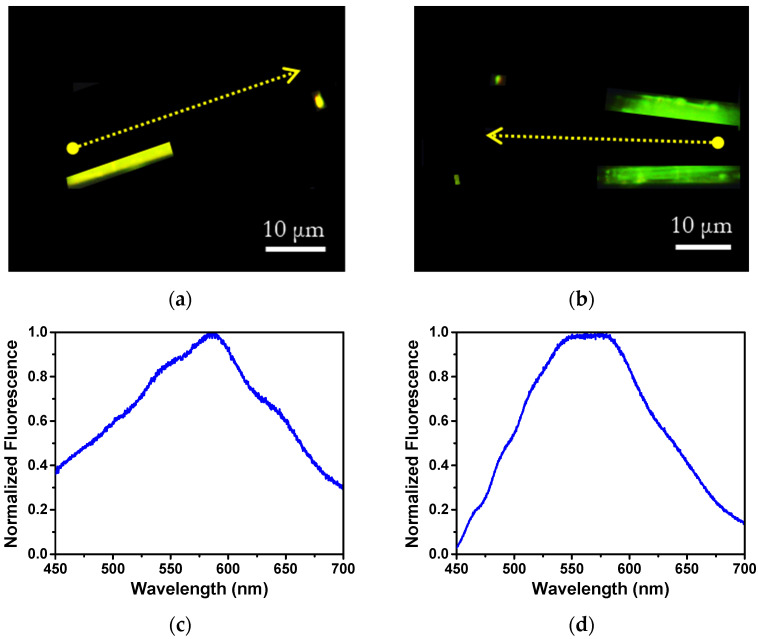
Photoluminescence microscopy images of (**a**) **1b** and (**b**) **1c**. The yellow-dotted arrows indicate the direction of light propagation. PL spectra of fibers (**c**) **1b** and (**d**) **1c**.

Crystals of **1b** obtained in CHCl_3_/CH_3_CN (Figure 7a) and **1c** in THF/ethanol (Figure 7b) showed bright yellow and green fluorescence, respectively. The PL spectra of **1b** (Figure 7c) and **1c** (Figure 7d) crystals were rather broad, with maxima at 586 nm and 564 nm, respectively. The bright emission observed at the fiber tip was motivated by outcoupled photons, due to the refractive index mismatch at the crystal/air interface, and confirmed that luminescence was efficiently waveguided along the fiber axis. 

In both compounds, a bathochromic shift of the emission bands was observed in the solid state with respect to solution motivated by the enhanced dielectric constant in the solid medium, as well as by intermolecular interactions [32]. Moreover, in both solid state and in solution, **1b** showed a larger red shift than **1c**, indicating the influence of the donor group in both states.

Next, we evaluated the optical loss coefficient (OLC) of **1c** fibers only, as **1b** fibers were too small to be measured with our experimental setup capabilities. 

To estimate the OLC of **1c**, a representative crystal of length ≈ 450 μm was studied. For this purpose, the crystal was excited with a 355 nm laser at different positions along the longitudinal axis and the resulting emission was detected at the fiber tip. As the light propagation distance increased, the spectra showed a progressive decrease in photoluminescence (PL) intensity caused by self-absorption, following the Beer–Lambert law, enabling the obtaining of the absorption coefficient (α) in μm^−1^.

**Figure 8:**
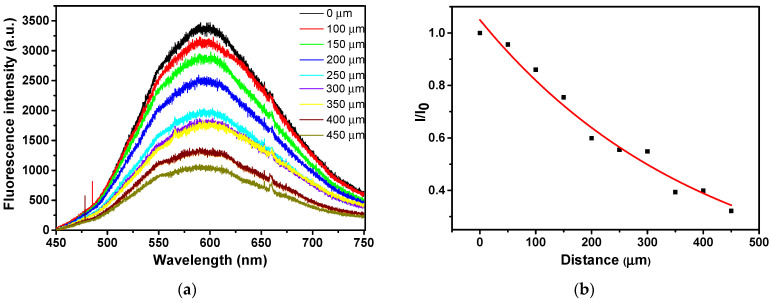
(**a**) PL spectra of the fiber collected at the tip upon varying the distances between the excitation and the tip of **1c**. Ratio between the PL intensities photoexcited at the tip (I_0_) and intermediate (I) positions along the fiber as a function of distance. (**b**) The red line in the right graph stands for fits to exponential laws of **1c**. In all the cases, crystal was photoexcited at 355 nm.

The OLC value was obtained after converting *α* to OLC in dB μm^−1^ (by using the relation OLC (dB μm^−1^) ≈ 4.34 *α (*μm^−1^)), yielding a 1.07 × 10^−2^ dB μm^−1^ for **1c** (Figure 8). This OLC value was much lower than we previously reported in other crystals with optical waveguide behavior [33] or in the order of the best derivatives reported recently [34,35].

### 2.6. X-ray Studies: Structure–Property Relationships

We have reported some structural requirements for supramolecular aggregates to behave as optical waveguides, adding a new dimension to the study of organic optical waveguides [34]. In this sense, the presence of internal channels should favor the propagation of light through the crystal. Furthermore, recent literature showed that the presence of microcavities within the crystals promotes the strong coupling between excitons and polaritons, resulting in the formation of exciton polaritons (Eps) that are responsible for light propagation [36]. 

Consequently, in order to further deepen the knowledge of the structure/property relationship in optical waveguides, an X-ray study of the **1b**,**c** crystals structures was carried out. 

Unfortunately, only the crystal structure of **1b** was successfully resolved by single crystal X-ray diffraction. Compound **1b** crystallized in the Pī space group and two molecules of **1b** and two molecules of acetonitrile used for crystallization completed the packing of the unit cell.

**Figure 9:**
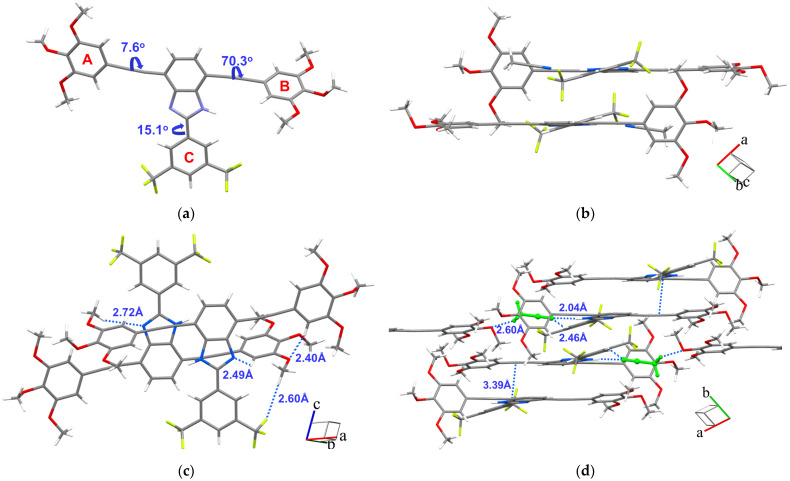
(**a**) Single-crystal X-ray structure of **1b**. (**b**) Front and (**c**) top view of the packing of the unit cell. (**d**) Molecular packing in the crystal of **1b**. Grey atoms are C, red atoms are O, blue atoms are N, yellow atoms are F and white atoms are H. Blue dotted lines show the non-covalent interactions.

The conformation of **1b** was non-planar, as the peripheral rings were rotated (7.3° for the A-ring, 70.3° for the B-ring and 15.1° for the C-ring) with respect to the 1*H*-benzo[*d*]imidazole core (Figure 9a). Therefore, **1b** molecules were arranged coplanar and antiparallel at 3.5 Å from each other (Figure 9b).

This arrangement was supported by hydrogen bonds between the pyridinic nitrogen and H*_ortho_* (2.49 Å) and with the methoxy groups of the B-ring (2.72 Å). Moreover, the methoxy groups of the B-ring interacted via hydrogen bond with the homologous groups of the A-ring (2.40 Å), as well as with the -CF_3_ groups (2.60 Å) (Figure 9c).

The formation of molecular layers was based on hydrogen bonds, where acetonitrile acted as a linker between **1b** molecules by interacting with the -NH group (2.04 Å) and the H*_ortho_* (2.46 Å) and methoxy groups of the A-ring (2.60 Å). C-H···π interactions between the triple bonds and the C-ring (3.39 Å) allowed the stacking of the molecular layers (Figure 9d). 

**Figure 10:**
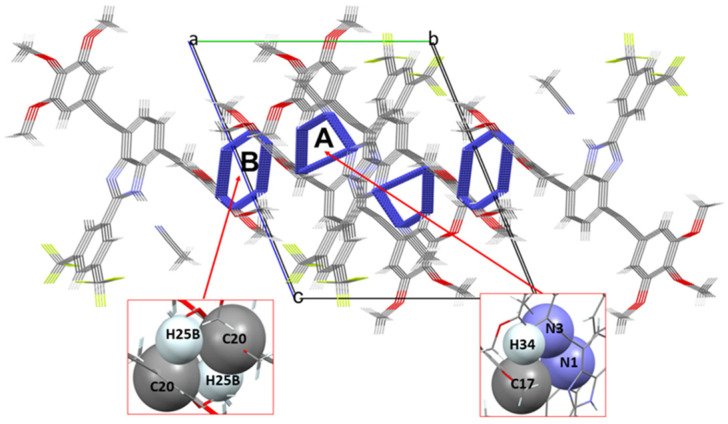
View down *a* axis of A and B channels for compound **1b**. The inset image shows the atoms that delimit the size of each channel with their Van der Waals radii. Grey atoms are C, red atoms are O, blue atoms are N, green atoms are F and white atoms are H.

In the three-dimensional arrangement of **1b**, two types of channels (A and B) extending in the direction of the *a* axis were observed. As shown in Figure 10, channels A were located inside the unit cell and channel B was located on the edges of the unit cell.

The formation of channel A can be attributed to hydrogen-bonding interactions between the two molecules that complete the packing of the unit cell (Figure S2, red lines). On the other hand, formation of channel B was exclusively due to the presence of C-H^…^π interactions between a C26 methoxy group and 3,5-bis(trifluoromethyl)phenyl ring, which occurred along the *b* axis (Figure S2, blue lines). 

The extension of the channels along the *a* axis and perpendicularly to the longest axis of growth (*c* axis) occurred due to the π^…^π interactions between 3,5-bis(trifluoromethyl)phenyl and 3,4,5-(trimethoxy)phenyl rings, with a distance of 4.05 Å between their planes (Figure S3).

These results confirmed that the presence of internal channels located in the direction perpendicular to the longest axis of growth allows the propagation of the light, supporting our previously reported hypothesis [34].

## 3. Materials and Methods

### 3.1. Materials and Reagents

The reagents needed for the synthesis of all the products described in this article that were commercially available were used with any kind of prior purification. All the air-sensitive reactions, such as the formation of the stannanes or the Still cross coupling reactions, were performed employing an argon atmosphere, making three vacuum-argon cycles. Synthesis in which microwave irradiation was the energy source was performed in a microwave Discover^®^ (CEM, Matthews, NC, USA).

NMR spectra (^1^H and ^13^C) were performed on Bruker Advance Neo NMR spectrometers working at 399.77 and 500.16 MHz for ^1^H and 100.53 and 125.75 MHz for ^13^C, respectively. The spectra were taken at 298 K in all the cases, employing partially deuterated solvents as the internal reference. The coupling constants (J) were described in Hertz (Hz) and chemical shifts (δ) in ppm. Signals’ multiplicities are noted as s = singlet, d = doublet, t = triplet, q = quadruplet, quint = quintuplet, sext = sextet, sept = septet, and m = multiplet.

For the photophysical studies, UV–visible studies were performed in a Jasco V-750 spectrophotometer (JASCO-Spain, Madrid, Spain) and the fluorescence studies were performed in a Jasco FP-8300 spectrofluorometer (JASCO-Spain, Madrid, Spain). The absorption and emission spectra shown in the main text were obtained from 10^−5^ M chloroform (with high spectroscopic grade) solutions at room temperature employing standard quartz cells with a width of 1 cm.

In order to determine the melting points, a Büchi model M-569 melting point meter was employed.

Regarding mass spectroscopy, MALDI-TOF mass spectra were employed using a Bruker Autoflex II TOF/TOF spectrometer with the employment of a matrix of dithranol, employing the positive mode. All the samples, which were co-crystallized with the matrix on the probe, were then ionized via a nitrogen laser pulse of 337 nm and accelerated under 20 Kv with time-delayed extraction before entering the time-of-flight mass spectrometer. Matrix (10 mg/mL) and sample (1 mg/mL) were separately dissolved in tetrahydrofuran (they are soluble in this solvent) and mixed in a different matrix/sample ratio range from 100:1 to 50:1. As is typical in these kinds of measurements, a 5 mL mixture of matrix and sample was applied to a MALDI-TOF MS probe and dried later under air. In addition, external calibration was carried out with the employment of Peptide Calibration Standard II (covered mass range: 700–3200 Da) from Care (Bruker, Billerica, MA, USA). The applied peak (*m*/*z* determination) detection method was the threshold centroid at 50% height of the maximum peak.

SEM images to determine the morphology of the supramolecular aggregates were obtained from a SEM Zeiss Gemini SEM 500 (Carl Zeiss AG, Oberkochen, Germany), which operated at 3 kV. The different crystals of all the derivatives, obtained by the slow diffusion technique, were deposited onto a glass substrate and the remaining solvent was slowly evaporated, without any further treatment.

X-ray diffraction data were extracted from a Bruker X8 APEX II CCD-based diffractometer (Bruker, Billerica, MA, USA). Data were processed using SAINT and, after that, an absorption correction was performed with the program SADABS. The structures were solved mixing direct methods and difference Fourier syntheses and, finally, refined by full-matrix least-squares on F2 employing the WINGX version 2021.3 and OLEX2-1.5 software packages. The rest of the atoms, except hydrogen, were refined with anisotropic thermal parameters. Hydrogen atoms were placed using a “riding model” and were included in the refinement at calculated positions.

To study the optical waveguiding behavior, PL microscopy images were taken from a Nikon Eclipse Ti inverted microscope (Nikkon Corporation, Tokyo, Japan) with dry objectives (100× N.A. 0.8 and 20× N.A. 0.45) coupled to a Shamrock spectrometer from Andor Technology (Andor, Belfast, UK) with a thermoelectrically cooled Newton EM (Andor) CCD. The excitation was obtained taking into account an appropriate filtering of the lines from a Xe lamp.

Finally, OLC to determine the efficiency of the optical waveguides of the fibers were calculated based on the excitation of the fibers using a pulsed Nd:YAG laser with the following characteristics: 355 nm, 300 ps, 1 KHz, and 30 μJ/pulse. Furthermore, a set of different filters were employed to attenuate the possible photoexcitation. The detection from the fiber edge was focused in free space on to a 0.5 m length SP2558 Princeton Instruments (Teledyne Acton Optics, Acton, MA, USA) spectrometer equipped with a 600 lines/mm grating and a liquid nitrogen-cooled CCD. 

### 3.2. Synthesis

#### 3.2.1. Synthesis of 2-(3,5-Bis(trifluoromethyl)phenyl)-4,7-dibromo-1H-benzo[d]imidazole (**4**) 

Compound **4** was synthesized as described by Murali and col [37]. To a mixture of compound **2** (0.1 g, 0.38 mmol) and 3,5-bis(trifluoromethyl)benzaldehyde (**3**) (0.091 g, 0.38 mmol) in DMF (10 mL), *p*-toluenesulfonic acid (0.015 g, 0.07 mmol) was added. The mixture was stirred for 1 h at 80 °C. The crude was poured into a 0.05 M aq. Na_2_CO_3_ (20 mL). The precipitate was filtered off and washed with water to obtain **4** as a brown solid (0.15 g, 84%). M.p.: 234–236 °C. ^1^H-NMR (CDCl_3_, ppm) δ: 13.76 (s, 1H, NH), 8.98 (s, 2H, H_2,6_-Ar), 8.32 (s, 1H, H_4_-Ar), 7.46 (s, 2H, H_5,6_). ^13^C-NMR (CDCl_3_, ppm) δ: 149.5 (C_2_), 142.9 (C_3a,7a_), 132.5 (q, *J_C-F_ =* 32.4 Hz, C_3,5_-Ar), 131.3 (C_1_-Ar), 127.5 (C_5,6_), 127.0 (q, *J_C-F_ =* 5.2 Hz, C_2,6_-Ar), 123.9 (sept, *J_C-F_ =* 3.6 Hz, C_4_-Ar), 122.9 (q, *J_C-F_ =* 273.0 Hz, CF_3_), 112.9 (C_4,7_). MS, calculated for C_15_H_6_Br_2_F_6_N_2_ M^+^ 485.88, was found to be 487.05.

#### 3.2.2. Procedure for the Synthesis of Arylakynylstannanes **6**

Arylethynylstannane **6a** is commercially available and was used without further purification. From **5b**–**c**, the arylethynylstannanes **6b**–**c** were synthesized, as previously reported [38]. 

General procedure: To a solution of the corresponding acetylene derivative (**5b**–**c**) (2 mmol) in dry THF (−78 °C) and under inert atmosphere, n-BuLi (1.87 mL, 3 mmol) was added slowly dropwise. Then, the mixture was stirred for 30 min. and, at that moment, Bu_3_SnCl (0.64 mL, 2.4 mmol) was added dropwise. The reaction mixture was allowed to warm until reaching room temperature and stirred again for 2 h. Then, to the crude reaction, 30 mL of water was added, neutralized with HCl 1M, and the organic layers were extracted with dichloromethane (3 × 30 mL). The organic layers were dried with MgSO_4_, the solvent was removed in vacuo, and the products were employed in the next step without further purification.

- Synthesis of tributyl((3,4,5-trimethoxyphenyl)ethynyl)stannane (**6b**)

From 5-ethynyl-1,2,3-trimethoxybenzene (**5b**) (0.38 g, 2 mmol), a yellow liquid was obtained (0.94 g, 97%). ^1^H-NMR (CDCl_3_, ppm) δ: 6.67 (s, 2H, H_2,6_-Ar), 3.84 (s, 3H, OCH_3_-3,5), 3.83 (s, 6H, OCH_3_-4), 1.61 (quint, *J* = 7.6 Hz, 6H, CH_2_-CH_2_-CH_2_), 1.38 (sext, *J* = 7.6 Hz, 6H, CH_2_-CH_2_-CH_3_), 1.07 (t, *J* = 7.6 Hz, 6H, Sn-CH_2_), 0.93 (t, *J* = 7.6 Hz, 9H, CH_3_).

- Synthesis of (benzo[*b*]thiophen-2-ylethynyl)tributylstannane (**6c**)

From 2-ethynylbenzo[*b*]thiophene (**5c**), (0.31 g, 2 mmol), a brown liquid was obtained (0.81 g, 91%). ^1^H-NMR (CDCl_3_, ppm) δ: 7.73 (m, 2H, H_4,7_), 7.51 (s, 1H, H_3_), 7,35 (m, 2H, H_5,6_), 1.62 (quint, *J* = 7.6 Hz, 6H, CH_2_-CH_2_-CH_2_), 1.38 (sext, *J* = 7.6 Hz, 6H, CH_2_-CH_2_-CH_3_), 1.07 (t, *J* = 7.6 Hz, 6H, Sn-CH_2_), 0.93 (t, *J* = 7.6 Hz, 9H, CH_3_).

#### 3.2.3. Procedure for the Synthesis of 2-(3,5-Bis(trifluoromethyl)phenyl)-4,7-bis(arylalkynyl)-1H-benzo[d]imidazoles **1**

A mixture of **4** (0.12 g, 0.25 mmol), the corresponding ethynylstannane **6** (0.62 mmol), PdCl_2_(PPh_3_)_2_ (0.007 g, 0.01 mmol) and LiCl (0.03 g, 0.75 mmol) was charged under inert atmosphere to a microwave vessel. CH_3_CN (1 mL) was added, the vessel was closed and irradiated at 110 °C for 20 min. The crude reaction was purified by column chromatography on silica gel, eluting with hexane/ethyl acetate to afford the pure products **1a**–**c**.

- Synthesis of 2-(3,5-bis(trifluoromethyl)phenyl)-4,7-bis(phenylethynyl)-1*H*-benzo[*d*]imidazole (**1a**)

From tributyl(phenylethynyl)stannane (**6a**) (0.24 g, 0.62 mmol), **1a** was obtained as a yellow solid (0.11 g, 87%) by chromatography, eluting with hexane/ethyl acetate 9:1. M.p.: 224–225 °C. ^1^H-NMR (CDCl_3_, ppm) δ: 10.20 (s, 1H, NH), 8.61 (s, 2H, H_2,6_-Ar_2_), 7.96 (s, 1H, H_4_-Ar_2_), 7.55 (m, 6H, 2 × H-Ph_2,6_, H_5,6_), 7.38 (m, 6H, 2 × H_3,4,5_-Ph). ^13^C-NMR (CDCl_3_, ppm) δ: 149.0 (C-2), 132.4 (q, *J_C-F_ =* 32.5 Hz, C_3,5_-Ar_2_), 131.8, 131.5 (2 × C_2,6_-Ph), 128.8, 128.6 (2 × C_3,5_-Ph), 128.4, 127.5 (2 × C_4_-Ph), 127.1, 127.0 (2 × C_1_-Ph), 123.7 (q, *J_C-F_ =* 5.2 Hz, C_2,6_-Ar_2_), 123.0 (q, *J_C-F_ =* 272.3 Hz, CF_3_), 122.6 (sept, *J_C-F_ =* 4.6 Hz, C_4_-Ar_2_), 95.7 (≡C-BIm), 85.2 (≡C-Ph). MS, calculated for C_31_H_16_F_6_N_2_ M^+^ 530.12, was found to be 531.18.

- Synthesis of 2-(3,5-bis(trifluoromethyl)phenyl)-4,7-bis((3,4,5-trimethoxyphenyl)ethynyl)-1*H*-benzo[*d*]imidazole (**1b**)

From tributyl((3,4,5-trimethoxyphenyl)ethynyl)stannane (**6b**) (0.30 g, 0.62 mmol), **1b** was obtained as a yellow solid (0.12 g, 70%) by chromatography, eluting with hexane/ethyl acetate 2:1. M.p.: 188–190 °C. ^1^H-NMR (CDCl_3_, ppm) δ: 11.33 (s, 1H, NH), 8.74 (s, 2H, H_2,6_-Ar_2_), 7.92 (s, 1H, H_4_-Ar_2_), 7.52 (s, 2H, H_5,6_), 6.82 (s, 2H, H_2,6_-Ar), 6.65 (s, 2H, H_2,6_-Ar), 3.85 (s, 12H, OCH_3_-3,5), 3.84 (s, 6H, OCH_3_-4). ^13^C-NMR (CDCl_3_, ppm) δ: 153.0 (C_2_), 149.4 (C_3,5_-Ar), 143.9 (C_3a,7a_), 135.6 (C_2_-Ar_2_), 132.5 (q, *J_C-F_ =* 32.4 Hz, C_3,5_-Ar_2_), 131.6 (C_4_-Ar), 127.7, 127.2 (C_5,6_), 123.6 (q, *J_C-F_ =* 5.2 Hz, C_2,6_-Ar_2_), 122.9 (q, *J_C-F_ =* 272.3 Hz, CF_3_), 122.3 (sept, *J_C-F_ =* 3.9 Hz, C_4_-Ar_2_), 117.4 (C_1_-Ar), 109.7 (C_2,6_-Ar), 95.0 (≡C-BIm), 83.4 (≡C-Ar), 60.9, 56.2 (6 × CH_3_). MS, calculated for C_37_H_28_F_6_N_2_O_6_ M^+^ 710.19, was found to be 711.52.

- Synthesis of 4,7-bis(benzo[*b*]thiophen-2-ylethynyl)-2-(3,5-bis(trifluoromethyl)phenyl)-1*H*-benzo[*d*]imidazole (**1c**)

From (benzo[*b*]thiophen-2-ylethynyl)tributylstannane (**6c**) (0.28 g, 0.62 mmol), **1c** was obtained as a yellow solid (0.09 g, 53%) by chromatography, eluting with hexane/ethyl acetate 9:1. M.p.: 215–216 °C. ^1^H-NMR (CDCl_3_, ppm) δ: 10.60 (s, 1H, NH), 8.67 (s, 2H, H_2,6_-Ar_2_), 7.97 (s, 1H, H_4_-Ar_2_), 7.80 (m, 4H, H_4,7_-BT), 7.55 (m, 4H, H_3_-BT, H_5,6_), 7.41 (m, 4H, H_5,6_-BT). ^13^C-NMR (CDCl_3_, ppm) δ: 150.8 (C-2), 144.2, 140.3 (C_3a,7a_), 139.4 (C_1_-Ar_2_), 132.3, 131.5 (H_3a,7a_-BT), (q, *J_C-F_ =* 32.5 Hz, C_3,5_-Ar_2_), 130.4, 128.1, 127.1, 126.7, 125.7, 124.8 (C_5,6_, C_2,3,4,5,6,7_-BT), 124.2 (q, *J_C-F_ =* 5.1 Hz, C_32,6_-Ar_2_), 123.7 (q, *J_C-F_ =* 273.2 Hz, CF_3_), 122.8 (sept, *J_C-F_ =* 3.9 Hz, C_4_-Ar_2_), 114.2, 107.4 (C_4,7_), 92.8 (≡C-BIm), 89.0 (≡C-BT). MS, calculated for C_35_H_16_F_6_N_2_S_2_ M^+^ 642.07, was found to be 643.48.

## 4. Conclusions

In this article, we described a new series of different symmetrical D–A–D 4,7-bis(arylethynyl)-1*H*-benzo[*d*]imidazole derivatives, introducing different donors and acceptors into the structure to modulate the energy value of the HOMO–LUMO gap. 

SEM images of the assembled crystals indicated that **1b** and **1c** derivatives formed needle-shaped crystals, suitable for application as optical waveguides. 

The PL microscopy studies revealed that **1b** and **1c** showed strong yellow-to-green luminescence with PL maxima around 586 and 564 nm, respectively. In addition, **1c** showed a low OLC, exhibiting an efficient light propagation. Thus, compounds **1b** and **1c** are the first reported 1*H*-benzo[*d*]imidazole derivatives to be applicable as optical waveguides.

Finally, the excellent morphology of an aggregate of **1b** allowed its study by X-ray diffraction. An in-depth analysis of the crystal structure verified that the existence of internal channels inside the crystal allows the transmission of light for the optical waveguiding behavior. These results are in agreement with previous X-ray diffraction studies on structures reported by our research group, confirming the first approximation of the structure/optical waveguide properties relationship.

## Figures and Tables

**Table 1:** Photophysical properties of **1a**–**c** (298 K, CHCl_3_, 10^−5^ M).

Compound	λ_abs_ (nm)	λ_em_ (nm)	Φ ^1^
**1a**	304/342	429	0.55
**1b**	284/353	459	0.77
**1c**	316/384	431, 455	0.78

^1^ PL quantum yields were measured in CHCl_3_ using quinine sulfate in H_2_SO_4_ 0.1 M (Φ = 0.54) and 9,10-diphenylanthracene in cyclohexane (Φ = 0.90) as internal standards.

## Data Availability

Not applicable.

## Supplementary Materials

The following supporting information can be downloaded at: https://www.mdpi.com/article/10.3390/molecules28124631/s1; Materials and reagents and procedures for the synthesis. Figure S1: UV-Vis absorption of derivatives **1** computed at the M06-2X/6-311+G(2d,p) level. Solvent effects were estimated using the polarizable continuum model (PCM) within the self-consistent reaction field (SCRF) approach using chloroform (ε = 4.7113) as solvent; Table S1: Photophysical properties of **1a-c** computed at the M06-2X/6-311+G(2d,p) level; Table S2: Crystal data and structure refinement for **1b**. Figure S2: View of packing for compound **1b** showing hydrogen bonding (red lines) and CH^…^π (blue lines) interactions; Figure S3: View of π^…^π and C-H^…^π interactions along *a* axis for compound **1b**.
