# Supplementary material for: Donor–Acceptor–Donor 1H-Benzo[d]imidazole Derivatives as Optical Waveguides
Source: Molecules. 2023 Jun 8;28(12):4631. doi: 10.3390/molecules28124631 (PMC10304989; doi:10.3390/molecules28124631)
Supplement: Supplementary file 1 [file molecules-28-04631-s001.zip › molecules-2401183-supplementary.pdf]

Supplementary Materials

Donor-Acceptor-Donor 1*H*-benzo[*d*]imidazole Derivatives as Optical Waveguides

Carlos Tardío [1], Javier Álvarez Conde [2], Ana M. Rodríguez [1], Pilar Prieto [1], Antonio de la Hoz [1,*], Juan Cabanillas-González [2,*] and Iván Torres-Moya [3,*]

[1] Department of Inorganic, Organic Chemistry and Biochemistry, Faculty of Chemical Science and Technologies, University of Castilla-La Mancha-IRICA, 13071 Ciudad Real, Spain; carlos.tardio@uclm.es (C.T.); anamaria.rfdez@uclm.es (A.M.R.); mariapilar.prieto@uclm.es (P.P.)

[2] Madrid Institute for Advanced Studies, IMDEA Nanociencia, Calle Faraday 9, Ciudad Universitaria de Cantoblanco, 28049 Madrid, Spain; javier.alvarez@imdea.org

[3] Department of Organic Chemistry, Faculty of Chemical Sciences, Campus of Espinardo, University of Murcia, 30010 Murcia, Spain

* Correspondence: antonio.hoz@uclm.es (A.d.l.H.); juan.cabanillas@imdea.org (J.C.-G.); ivan.torres@um.es (I.T.-M.)

S1. Theoretical Calculations

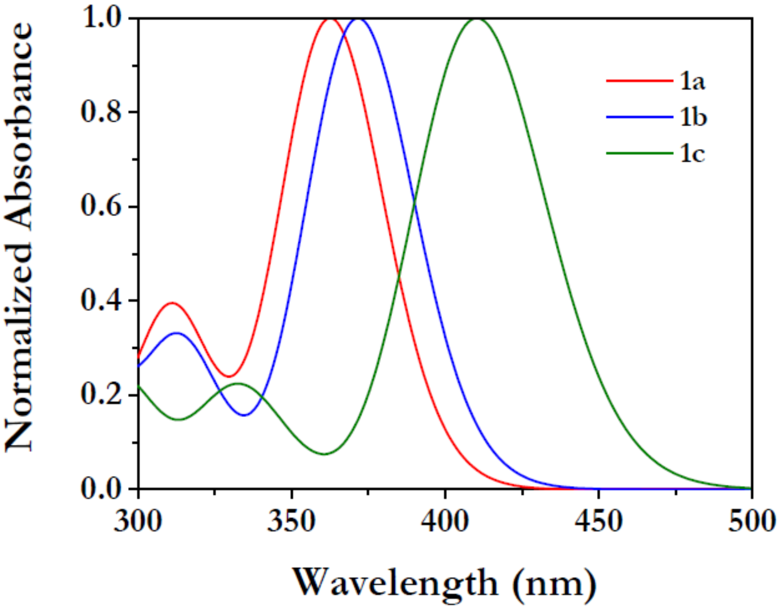

Figure S1. UV-Vis absorption of derivatives **1** computed at the M06-2X/6-311+G(2d,p) level. Solvent effects were estimated using the polarizable continuum model (PCM) within the self-consistent reaction field (SCRF) approach using chloroform (ε = 4.7113) as solvent.

Table S1. Photophysical properties of **1a-c** computed at the M06-2X/6-311+G(2d,p) level.

Compound	λ_{abs}	f	Description
**1a**	357	2.01	H→L (94%)
	313	0.68	H-1→L (87%)
**1b**	371	2.01	H→L (88%)
	313	0.64	H-4→L (49%), H-1→L (38%)
**1c**	409	2.60	H→L (90%)
	332	0.57	H-1→L (46%), H-3→L (21%)

S2. X-Ray Diffraction

Table S2. Crystal data and structure refinement for **1b**.

Empirical formula	C19.5H15.5F3N1.5O3
Formula weight	375.83
Temperature/K	293(2)
Crystal system	Triclinic
Space group	P$\bar{1}$
a/Å	10.77(2)
b/Å	12.65(3)
c/Å	14.33(3)
α/°	66.97(3)
β/°	83.86(3)
γ/°	77.09(3)
Volume/Å3	1750(7)
Z	4
ϱcalc g/cm3	1.426
μ/mm-1	0.118
F(000)	776.0

Crystal size/mm3	0.10 × 0.09 × 0.04
Radiation	MoKα (λ = 0.71073)
2Θ range for data collection/°	4.724 to 49.996
Index ranges	-12 ≤ h ≤ 12, -15 ≤ k ≤ 15, -16 ≤ l ≤ 17
Reflections collected	10758
Independent reflections	5987 [Rint = 0.1755, Rsigma = 0.3271]
Data/restraints/parameters	5987/96/550
Goodness-of-fit on F2	0.953
Final R indexes [I≥2σ (I)]	R1 = 0.1204, wR2 = 0.2337
Largest diff. peak/hole / e Å-3	0.46/-0.39

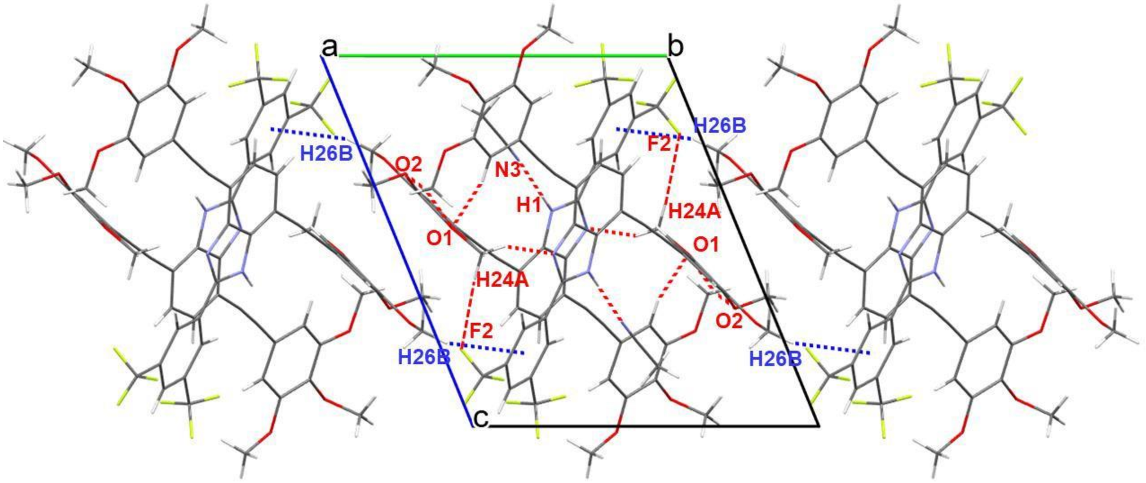

Figure S2. View of packing for compound **1b** showing hydrogen bonding (red lines) and CH···π (blue lines) interactions.

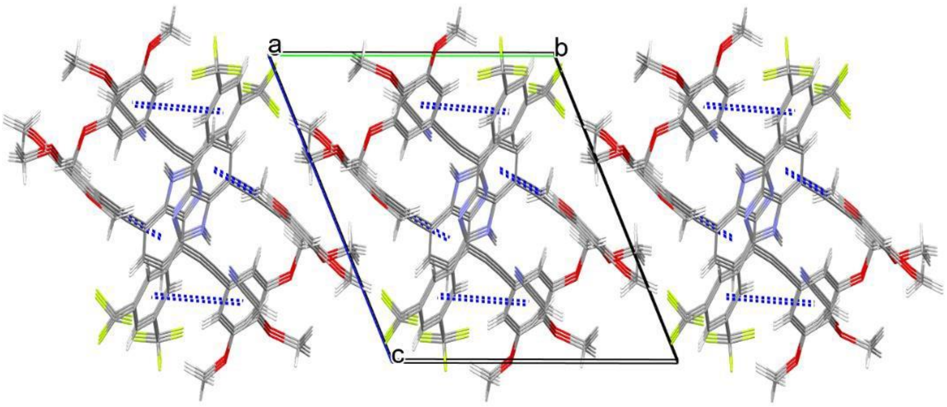

Figure S3. View of π···π and C-H···π interactions along *a* axis for compound **1b**.
